# Complete *Anopheles funestus* mitogenomes reveal an ancient history of mitochondrial lineages and their distribution in southern and central Africa

**DOI:** 10.1038/s41598-018-27092-y

**Published:** 2018-06-13

**Authors:** Christine M. Jones, Yoosook Lee, Andrew Kitchen, Travis Collier, Julia C. Pringle, Mbanga Muleba, Seth Irish, Jennifer C. Stevenson, Maureen Coetzee, Anthony J. Cornel, Douglas E. Norris, Giovanna Carpi

**Affiliations:** 1 Department of Molecular Microbiology and Immunology, Johns Hopkins Malaria Research Institute, Johns Hopkins Bloomberg School of Public Health, Baltimore, MD USA; 2 University of California at Davis, Davis, CA USA; 3 Department of Anthropology, University of Iowa, Iowa City, IA USA; 4 Daniel K. Inouye US Pacific Basin Agricultural Research Center (PBARC), Department of Agriculture, Agricultural Research Service, Hilo, Hawaii USA; 5 Tropical Diseases Research Centre, Ndola, Zambia; 6 U.S. President’s Malaria Initiative and Centers for Disease Control and Prevention, Atlanta, Georgia; 7 Macha Research Trust, Choma, Zambia; 8 Wits Research Institute for Malaria and Wits/MRC Collaborating Centre for Multidisciplinary Research on Malaria, School of Pathology, University of the Witwatersrand, Johannesburg, South Africa; 9 Centre for Emerging Zoonotic and Parasitic Diseases, National Institute for Communicable Diseases, Johannesburg, South Africa

## Abstract

*Anopheles funestus s*.*s*. is a primary vector of malaria in sub-Saharan Africa. Despite its important role in human *Plasmodium* transmission, evolutionary history, genetic diversity, and population structure of *An*. *funestus* in southern and central Africa remains understudied. We deep sequenced, assembled, and annotated the complete mitochondrial genome of *An*. *funestus s*.*s*. for the first time, providing a foundation for further genetic research of this important malaria vector species. We further analyzed the complete mitochondrial genomes of 43 *An*. *funestus s*.*s*. from three sites in Zambia, Democratic Republic of the Congo, and Tanzania. From these 43 mitogenomes we identified 41 unique haplotypes that comprised 567 polymorphic sites. Bayesian phylogenetic reconstruction confirmed the co-existence of two highly divergent *An*. *funestus* maternal lineages, herein defined as lineages I and II, in Zambia and Tanzania. The estimated coalescence time of these two mitochondrial lineages is ~500,000 years ago (95% HPD 426,000–594,000 years ago) with subsequent independent diversification. Haplotype network and phylogenetic analysis revealed two major clusters within lineage I, and genetic relatedness of samples with deep branching in lineage II. At this time, data suggest that the lineages are partially sympatric. This study illustrates that accurate retrieval of full mitogenomes of *Anopheles* vectors enables fine-resolution studies of intraspecies genetic relationships, population differentiation, and demographic history. Further investigations on whether *An*. *funestus* mitochondrial lineages represent biologically meaningful populations and their potential implications for malaria vector control are warranted.

## Introduction

In 2016, there were approximately 216 million cases of malaria and approximately half a million deaths, most of which occurred in sub-Saharan Africa^1^. These data represent a drastically reduced incidence of malaria since 2000^2^. However, progress has plateaued in recent years and incidence and mortality have remained essentially the same in 2015 and 2016^1^. The decline of malaria can be attributed to several causes, including a rise in coverage of vector control^1,2^. However, phenomena such as changing vector foraging and resting behaviors^3,4^, and the development of insecticide resistance^5,6^, have led to heterogeneity and stagnation in the success of malaria control worldwide. Population genetic and genomic methods, as a result of advances in sequencing strategies, are becoming useful tools for understanding and monitoring vector population diversity^7–9^, dispersal^10^ and dynamics with the ultimate goal of informing malaria control^11,12^.

*Anopheles funestus sensu stricto* (hereafter “*An*. *funestus*”) is a major malaria vector throughout sub-Saharan Africa and poses a significant threat to malaria control and elimination due to its high vectorial capacity, expansive distribution, and high rates of insecticide resistance^13^. While studies of the other major regional malaria vectors in the *An*. *gambiae sensu lato* species complex have been frequent over the past four decades, research on *An*. *funestus* has remained at a trickle, with very few investigations during recent decades. Notably, this dearth is largely due to the relative difficulty of rearing *An*. *funestus* in laboratories. With the advent of cytogenetic studies in the 1980’s, as well as modern and more sophisticated molecular techniques, population studies of field-caught mosquitoes have become more common^13^. Additionally, the establishment of laboratory colonies of *An*. *funestus* within the last decade^14^ has finally allowed for more complex genetic and genomic studies of this species^13,15^.

However, there are still only a limited number of genetic studies (using a variety of mitochondrial and nuclear markers) of *An*. *funestus* across its entire geographic range in sub-Saharan Africa^16–25^. These broad-scale studies largely agree that *An*. *funestus* populations can be split into major western and eastern groups^16,17^. Interestingly, there is compelling evidence for the hypothesis that the Great Rift Valley serves as an important barrier to gene flow between these populations^16–20^, which has similarly been documented for *An*. *gambiae*^26^. Additionally, Michel *et al*.^17^ reported two mitochondrial lineages (I and II) of *An*. *funestus* based on partial mitochondrial gene sequences (COI and ND5), which are not reflected in parallel nuclear microsatellite analyses. While mitochondrial lineage I makes up the majority of samples found in countries throughout sub-Saharan Africa, individuals belonging to lineage II have only been reported in the southeastern range of this species in Mozambique, Madagascar, northeastern Tanzania, and northern Zambia^17,23,25,27^.

To date, fine-scale population genetic studies of *An*. *funestus* have focused on only limited regions in western and eastern Africa, while *An*. *funestus* populations in central and southern Africa, such as Zambia and the Democratic Republic of the Congo, remain greatly understudied. More specifically, investigations of *An*. *funestus* maternal lineages have also been limited within southern Africa, and have solely relied on partial mitochondrial gene sequences (COI and ND5)^17,23–25^. A key challenge to such studies is the unfinished nature of the published mitochondrial reference genome (GenBank: DQ146364.1), which is incomplete and lacking ~27.5% of the genome sequence, mainly in coding regions^28^. This incomplete reference represents a hurdle to future research in the field, as mitochondrial genomes (hereafter “mitogenome”) can serve as an important source of markers for population genetic studies, and also provide insight into evolutionary relationships within the *An*. *funestus* species complex. Further, the absence of large-scale mitogenome and nuclear genome data of wild-caught *An*. *funestus* makes it difficult to catalogue genetic variation in natural populations and determine population structure and dispersal rates.

Complete mitogenomes are particularly useful for reconstructing phylogenies and inferring population history due to haploid maternal inheritance^29,30^, the rare occurrence of recombination^31^, and a higher mutation rate than the nuclear genome^32^. Mitochondrial sequence polymorphisms may be particularly useful to study sex-biased dispersal known to occur in some anopheline mosquitoes (including *An*. *funestus*)^33,34^. Mitogenomes have smaller effective population sizes than autosomal DNA, enabling better discrimination between populations due to the rapid effects of genetic drift. Additionally, mitochondria contain multiple genomic copies making mitogenomes amenable targets for sequencing at high coverage. Understanding historical gene flow and genetic structure via analysis of mitogenomes is a step toward revealing contemporary vector population dynamics and accurate discrimination between lineages and sub-populations. In turn, this information will contribute to an enhanced appreciation of malaria transmission dynamics, especially if vector genetic diversity reflects differences in biology, behavior, permissiveness to *Plasmodium* parasites^35^, or insecticide susceptibility^11,36^, all of which have consequences for malarial disease management, surveillance, and control measures.

To investigate the degree of genetic diversity in *An*. *funestus* across a critically understudied geographic region, and to further examine the evolutionary history and distribution of mitochondrial lineages, we performed shotgun mitogenome sequencing of *An*. *funestus* samples from northern Zambia, southeastern Democratic Republic of the Congo (hereafter “DRC”), and southern Tanzania. We first generated a new *An*. *funestus* mitochondrial reference genome that filled gaps in the existing incomplete reference (GenBank: DQ146364.1)^28^ and then assembled and annotated 43 *An*. *funestus* mitogenome sequences from these regional collections. Bayesian phylogenetic and classical population genetic analyses were performed to characterize *An*. *funestus* mitochondrial lineages, document their distribution in southern and central Africa, and assess their demographic history. Notably, the data generated in this study are part of an initial collection effort to build a digital repository of genomic data from field-caught *An*. *funestus* across southern and central Africa.

## Methods

### Study Sites and Sample Selection

*An*. *funestus* samples were chosen to geographically represent this species in southern and central Africa where we are actively conducting research on malaria transmission. In total, 43 *An*. *funestus* samples were selected for further sequencing (Table 1). Nchelenge District of northern Zambia was chosen as a long-standing site for malaria research in within the framework of “The Southern and Central Africa International Centers for Excellence in Malaria Research (ICEMR)”, which is a research program designed to understand drivers of persistently high malaria transmission. Nchelenge District abuts the Democratic Republic of the Congo, with the border bisecting Lake Mweru. The sampling area lies 807 meters above sea level, with a marsh ecotype and three seasons: a single rainy season from November to May, a cool dry season from May to August, and a hot dry season from August to November. Malaria transmission occurs at high rates year-round, despite widespread use of long-lasting insecticide treated nets (LLINs) and indoor residual spraying (IRS)^37,38^. Although both *An*. *funestus* and *An*. *gambiae* are present in this district, *An*. *funestus* is the primary vector in Nchelenge, with the population peaking during the dry season^39^.

**Table 1:** Sampling sites, methods, numbers (N) and collection dates for whole genome sequenced specimens.

Site	N	Country	Coordinates (lat., long.)	Method	Collection Date
Nchelenge	6	Zambia	−9.2869, 28.7590	Indoor CDC-LT	Feb, 2015
Nchelenge	5	Zambia	−9.3247, 28.7819	Indoor PSC	Apr, 2015
Nchelenge	6	Zambia	−9.3042, 28.7822	Indoor CDC-LT	Feb, 2015
Nchelenge	6	Zambia	−9.2926, 28.7539	Indoor PSC	Apr, 2015
Kilwa Island	5	Zambia	−9.2675, 28.4500	Indoor backpack aspiration	Aug, 2014
Kapolowe	5	DRC	−10.9398, 26.9530	Indoor HLC	Apr, 2015
Lupiro	5	Tanzania	−8.383, 36.667	Indoor backpack aspiration	Jun, 2013
Lupiro	5	Tanzania	−8.383, 36.667	Animal-pen backpack aspiration	Jun, 2013

Kapolowe is a town in Haut-Katanga Province, in southeastern Democratic Republic of the Congo. It is on the edge of Lake Tshangalele, an artificial lake created by the dam at nearby Mwadingusha. Kapolowe is at an elevation of 1,177 meters above sea level and has a rainy season lasting from November to April, with a dry season between May and October. Malaria prevalence is high despite widespread use of LLINs^40^ and no IRS has been conducted in Kapolowe. *Anopheles gambiae*, *An*. *funestus*, and *An*. *coustani* group mosquitoes are the most commonly collected anopheline mosquitoes in Kapolowe^41^.

Lupiro is located within Kilombero Valley in southern Tanzania, a zone of intense perennial malaria transmission^42–46^. It is at an elevation of 300 meters above sea level and has a rainy season lasting from November to May. Epidemiological studies in this valley have revealed that malaria transmission intensities are very high, with 100–1000 s of infective bites per person per annum^42,46–48^. A nation-wide LLIN distribution program is currently underway in Tanzania, through which net coverage has substantially increased in Kilombero Valley^49^. However, reduction in malaria transmission was not as great as anticipated based on the high LLIN coverage (75%) achieved^49^.

### DNA extraction and sequencing

Field-caught mosquitoes were morphologically identified to species using standard keys at the time of collection^50^. Each identified mosquito was placed individually into a labelled 0.6 mL microcentrifuge tube containing silica gel desiccant and cotton wool and stored either at room temperature or frozen at −20 °C until laboratory processing. Genomic DNA extractions were performed on the head and thorax for each individual mosquito as previously described^51^. Quantitation of the genomic DNA was performed using a Qubit 2.0 Fluorometer (Life Technologies, Grand Island, NY) and genomic libraries were prepared as described, using an input of 10 ng of genomic DNA^52^. Indexed libraries were pooled and sequenced in a single lane on an Illumina HiSeq4000 to generate 150 bp paired-end reads. Sequencing was performed at the University of California-Davis DNA Technologies Core. Demultiplexed Illumina raw reads obtained from DNA Technologies Core were trimmed using Trimmomatic version 0.36^53^. We used the typical trimming parameters “ILLUMINACLIP:’{input.adapters_file}’:2:30:10 LEADING:3 TRAILING:3 SLIDINGWINDOW:4:15 MINLEN:36”, which removes adapters, trims low quality or N bases below quality 3, scans the read with a 4-base sliding window cutting when the average quality per base drops below 15, and dropped reads below 36 bp long.

### Mitochondrial genome assembly and variant detection

The incomplete *An*. *funestus* mitochondrial reference (GenBank: DQ146364.1)^28^ was used as a ‘seed’ sequence to generate a new and complete mitogenome reference with MITObim v1.8 with 10 iterations, default parameters, and trimmed Illumina reads from sample AF13ICNC14-106^54^. Subsequently, raw Illumina sequence reads for each sample were aligned to the newly generated *An*. *funestus* mitogenome reference AF13ICNC14-106, using BWA alignment tool v0.7.7 (bwa-mem, default parameters)^55,56^. Duplicate sequences were identified and excluded from downstream analysis using Picard Suite v1.117 MarkDuplicates^57^. Aligned reads were realigned around indels (insertions and deletions) using GATK v3.7 RealignerTargetCreator and IndelRealigner. Variants with respect to AF13ICNC14-106 were identified with GATK HaplotypeCaller (ploidy set to 1)^58^. Indels and single nucleotide polymorphisms (SNPs) with signals of low mapping or genotyping quality were excluded with GATK VariantFiltration, using the following filters recommended by GATK: quality by depth (QD < 2.0), Fisher strand bias (FS > 200.0), mapping quality (MQ < 40.0), the Mann-Whitney rank sum test (ReadPosRankSum < − 20.0)^59^. To create the consensus mitogenome sequence for each sample from the variant files, the GATK tool FastaAlternateReferenceMaker was used. The mitogenome coverage for each sample was calculated using the software GATK v3.7 (DepthOfCoverage with parameters mmq > 20 and mbq > 20)^58^.

### Phylogenetic analysis and divergence time estimation

The 43 newly generated *An*. *funestus* mitogenomes were aligned using MUSCLE with and without full mitogenomes from *An*. *gambiae* (GenBank: L20934.1) and *An*. *minimus* (GenBank: KT895423.1) as outgroups^60–62^. It is important to account for recombination when reconstructing evolutionary histories because homologous recombination has a profound impact on evolutionary trajectories and therefore the interpretation of inferred phylogenies. We used the 3SEQ software which implements a fast non-parametric recombination detection algorithm to infer recombinant tracts along the mitogenomes to rule out the possibility of recombination in our N = 43 *An*. *funestus* mitogenome alignment^63^. Maximum likelihood trees of the 43 samples were generated in SeaView v4, using PhyML and GTR substitution model, and default parameters with 1000 bootstrap replicates^64^. BEAST2 v2.4.5 was used to conduct phylogenetic analyses as well as generate estimates of divergence times and population size, and determine demographic history of southern and central African *An*. *funestus*^65^. Analyses were performed using a substitution rate of 1.2 × 10^−8^ mutations per site per year, following estimates from Brower^66^. Markov chains were run for 100 million generations or until convergence, with 10 million generations of each run discarded as burn in, and chains sampled every 10000 generations. Both HKY and GTR substitution models were used in combination with gamma site-specific rate variation (G) and a proportion of invariant sites (I) parameters, strict and relaxed log normal molecular clocks, as well as constant and Bayesian skyline population models. To compare models, the Path Sampler application from BEAST2 v2.4.5 was used to generate marginal likelihood estimates and the model with the highest estimate was used for demographic and population history inference^65^. Tracer v1.6^67^ was used to inspect convergence and confirm effective sample sizes were greater than 200 for parameters of interest. Tracer v1.6 was also used to generate Bayesian skyline plots. Because our evolutionary rate was in years, effective population size was confounded with generation time; we used a generation time of 3/52^68^ to convert estimates of population diversity to N_e_ in our coalescent analyses. LogCombiner was used to resample 10000 trees from BEAST2 analysis and then TreeAnnotator was used to generate Maximum Clade Credibility (MCC) trees^65^. The multiple alignment of the 43 *An*. *funestus* mitogenomes was further analyzed using TCS statistical method as implemented in PopArt v1.7 to produce a mitochondrial haplotype network^69,70^.

To investigate how the genetic diversity of *An*. *funestus* samples sequenced in this study compared to previously known *An*. *funestus* diversity we extracted the NADH dehydrogenase subunit 5 (ND5) sequences from our 43 mitogenome sequences and aligned them using MUSCLE to 400 published partial ND5 sequences (834 bp)^17^. To further explore the diversity of our samples in the context of this large pan-African dataset, we used PhyML to generate a maximum likelihood tree of the ND5 alignment using the GTR nucleotide substitution model and 1000 bootstrap replicates^71^. tcsBU was used to visualize the TCS haplotype network generated by TCS v1.21 for partial ND5 sequences^69,72^.

### Phylogenetic Analysis of Geographic Structure

To determine the extent of geographic structure in our *An*. *funestus* populations, we estimated the strength of association between phylogenetic relationships and sampling locations for the complete *An*. *funestus* mitogenome sequences using the software package BaTS^73^. BaTS generates a parsimony score (PS)^74^ and association index (AI)^75^ to assess the extent of geographical association with phylogenetic structure across the entire tree, as well as maximum monophyletic clade size statistics (MC)^73^ to determine the association for particular sampling locations.

### Estimation of demographic history

DnaSP v5 was used to generate general diversity statistics, conduct neutrality tests, and examine demography^76^. These statistics test the null hypothesis that populations are: neutral, of constant size, are in panmixia, and have no recombination. Arlequin v3.5 was used to calculate the mismatch distributions to test signal for population spatial expansion^77^. The raggedness index and SSD were used to evaluate how well the sample conforms to the null model of either demographic or spatial expansion. Mantel tests were used to evaluate for correlation between genetic distance and physical distance using the APE package in R v3.3.0^78,79^.

### Annotation and data availability

Protein coding genes were identified and annotated manually by sequence similarity to the previous reference genome (GenBank: DQ146364.1) as well as the orthologous sequences of other anopheline species^28,61,62^. Transfer RNA (tRNAs) were identified by their putative secondary structures using tRNAscan-SE^80^. The ribosomal RNA genes (rrnL and rrnS) were identified by sequence similarity to the available homologous sequences using MITOS^81^.

The 43 newly generated *An*. *funestus* mitogenome sequences are available in the GenBank Database under the following accession numbers: MG742157-MG742199.

## Results

### *An. funestus* mitogenomes

A total of 43 female *An*. *funestus* from three regions across southern and central Africa (Zambia N = 28; Tanzania N = 10; DRC = 5) were subjected to whole genome shotgun sequencing (Table 1). From these data, the first complete *An*. *funestus* reference mitochondrial genome (15,353 bp in length) was generated and the remaining 42 mitogenomes were assembled. On average, mitogenome coverage was 350×, ranging from 32× to 477× across the 43 samples (Figure S1). The nucleotide composition of the *An*. *funestus* mitogenome reference was heavily AT-skewed (average AT content = 78.2%), as is typical for the mitogenomes of many arthropod and anopheline taxa^82^. The mean nucleotide diversity (π) in the 43 *An*. *funestus* mitogenomes was 0.00625 (SD ± 0.00054, Table 2), which is higher than the nucleotide diversity previously estimated using partial sequences of mitochondrial genes (π = 0.0042, SD ± 0.007)^17,83^, and that of other major anopheline malaria vectors, *An*. *gambiae* (π = 0.0038) and *An*. *arabiensis* (π = 0.0046)^84^. The multiple alignment of the 43 mitogenomes revealed a total of 567 polymorphic sites. These variable sites defined a total of 41 mitogenome haplotypes from the 43 sampled individuals (mean haplotype diversity = 0.998, SD: ± 0.006, Table 2), with only AF13ICNC14-155:AF15R31C10-A001 and AF15R35C07-B001:AF15R35C07-F002 sample pairs representing the same mitogenome haplotypes.

**Table 2:** Diversity statistics, neutrality tests, and demographic analysis.

	Total	Lineage I	Lineage II	DRC	Zambia	Tanzania
# Samples	43	29	14	5	28	10
# Haplotypes	41	28	13	5	26	10
H_d_ (sd)	0.998 (0.006)	0.998 (0.010)	0.989 (0.031)	1.000 (0.126)	0.995 (0.011)	1.000 (0.045)
π (sd)	0.00625 (0.00054)	0.00237 (0.00019)	0.00500 (0.00033)	0.00163 (0.00022)	0.00668 (0.00046)	0.00611 (0.00139)
K	95.93	36.320	76.725	25.000	102.571	93.756
**Neutrality Tests**						
Tajima’s D	−1.075	−1.841**	−1.040	−1.210	−0.514	0.023
Fu and Li’s D*	−1.912*	−2.385*	−0.908	−1.210*	−1.098	0.076
Fu and Li’s F*	−1.915	−2.606**	−1.086	−1.312	−1.069	0.071
Fu’s Fs	−4.278	−6.543	0.904	0.778	−0.514	0.0525
**Mismatch Distribution: Demographic Expansion**						
SSD	0.00947	0.0123**	0.0284**	0.126**	0.013**	0.0365
Raggedness index	0.00309	0.00909*	0.0156	0.300	0.00638	0.0415
**Mismatch Distribution: Spatial Expansion**						
SSD	0.00939**	0.0135	0.0202	0.118**	0.0103**	0.034
Raggedness index	0.00309	0.00909	0.0156	0.300	0.00638	0.0415

Samples have been split into two general comparisons: lineage I vs lineage II and DRC vs Tanzania vs Zambia. p-value is indicated by *0.10 > p > 0.05 or **p < 0.05.

### Phylogenetic analysis and divergence time estimation

**Figure 1:**
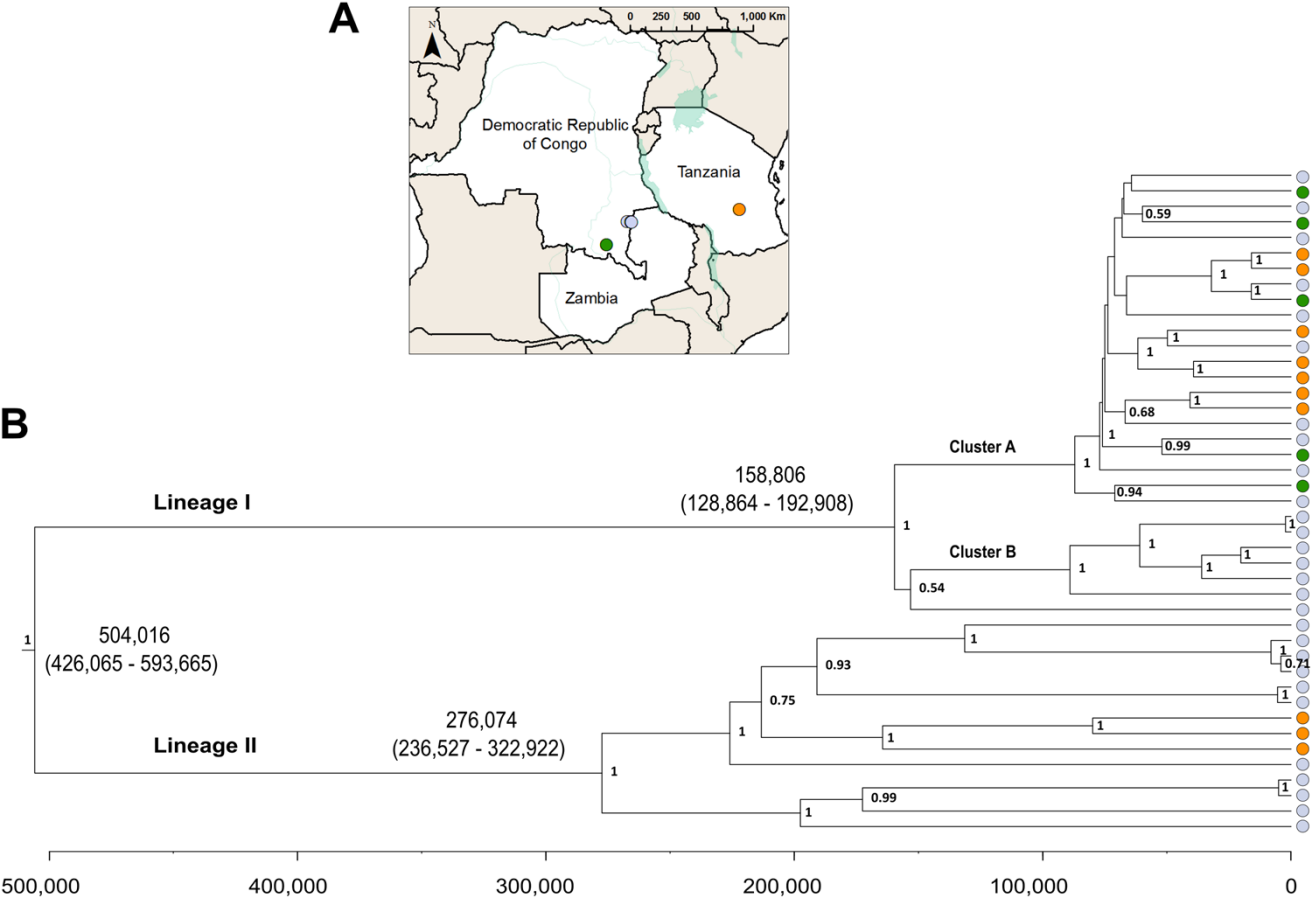
Map and phylogenestic relationships of 43 *An*. *funestus* mitogenomes. (**A**) Map indicating the collection sites for 43 *An*. *funestus* samples, created using ArcGIS v10.5.1 (www.esri.org). (**B**) Bayesian maximum clade credibility phylogeny of complete mitogenomes from the 43 *An*. *funestus* samples of the best fitting model (GTR + G + I, Bayesian skyline plot, and a relaxed molecular clock) inferred using BEAST2. Samples are colored by geographic origin: blue indicates Zambia (N = 28), orange indicates Tanzania (N = 10), green indicates DRC (N = 5). Divergence dates (median estimates and 95% HPD) are given in parenthesis for major nodes. Posterior probabilities > 0.5 are indicated at each node. The timescale is indicated below the tree and is in years before present.

To investigate the phylogenetic relationships of the 43 *An*. *funestus* mitogenomes sequenced in this study (Figure 1A), we constructed a maximum likelihood tree and identified two distinct lineages, herein defined as lineages I and II (Figure S2), which corresponded to clades 1 and 2 as described in Michel *et al*.^17^. Our Bayesian coalescent analysis, implemented in BEAST2, produced a tree with concordant topology to the ML tree (Figure 1B). The most frequently sampled lineage in our study, lineage I, included mitogenomes from all sampled sites in the three countries. Lineage II, on the other hand, was absent from our DRC collection, which may be due to the small sample size.

We found 160 nucleotide differences between haplotypes in lineage I and II on average, with 47 fixed differences between the two mitochondrial lineages. Lineage II contained longer branch-lengths between samples than samples within lineage I, an observation which is also reflected in the diversity statistics (Table 2). The most recent common ancestor between the two lineages was estimated to have existed 504,016 years ago (95% Highest Posterior Density (HPD): 426,065–593,665 ya). To validate the accuracy of divergence time estimations, we computed a separate BEAST2 analysis that included two outgroups, *An*. *gambiae* and *An*. *minimus*, in addition to our samples (see Figure S3). This analysis gave an approximate date of divergence between the two lineages of 528,336 years (95% HPD: 439,666–626,020). The divergence time of *An*. *gambiae* from all other anophelines in the analysis was 9.56 million years ago (95% HPD: 7.44–12.03 Mya), while *An*. *minimus* appears to have split from *An*. *funestus* approximately 5.36 million years ago (95% HPD: 4.06–6.68 Mya).

**Figure 2:**
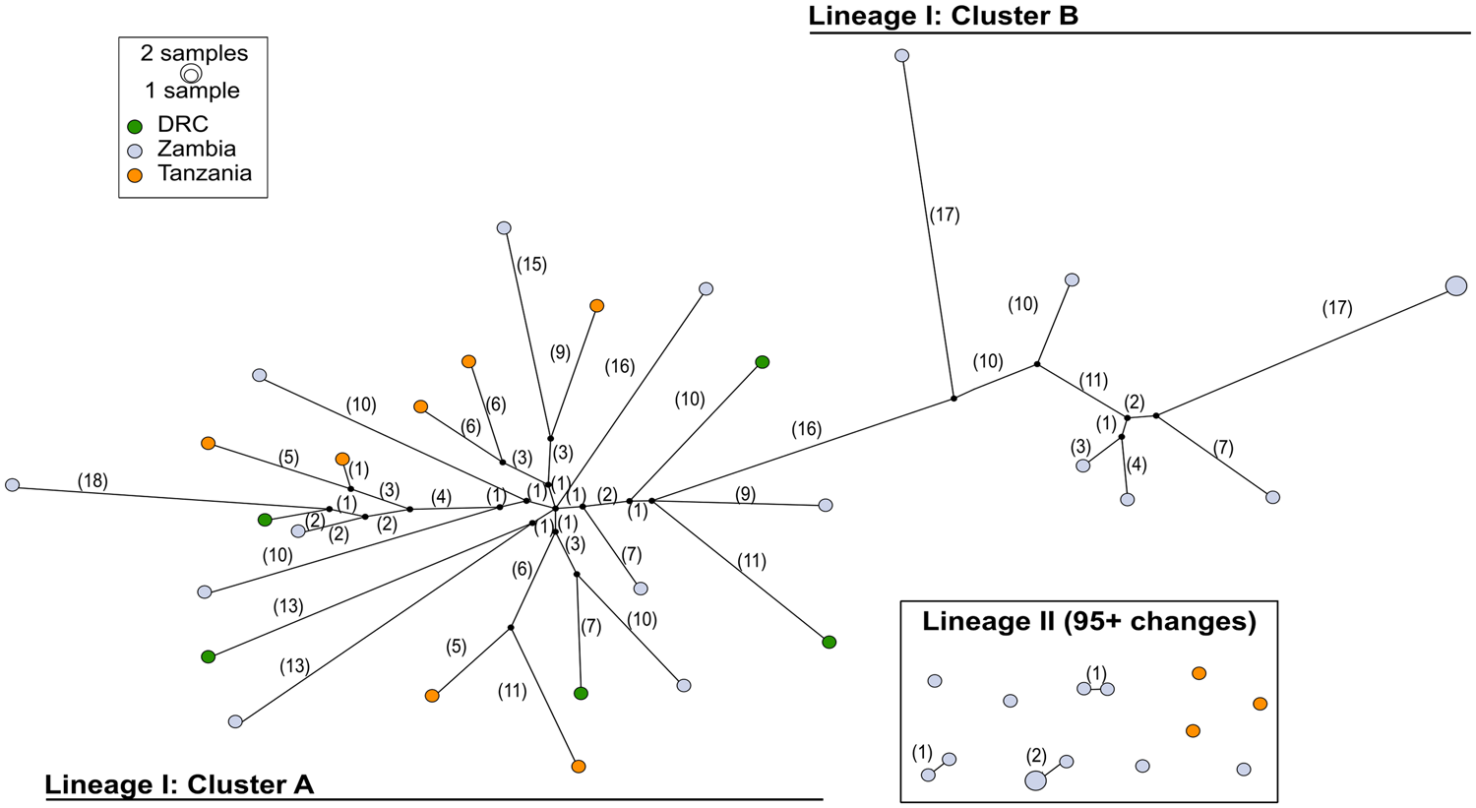
Haplotype network of 43 *An*. *funestus* mitogenomes. In this TCS network, each node indicates a haplotype, with nodes colored according to origin. The number of mutational steps between nodes are indicated in parentheses beside the line connecting one node to another. One group of samples (all lineage II) did not connect to the main cluster within 95 mutational steps (over a 95% confidence limit for connectivity): these are shown in the box in the lower right. There are two distinct groups within the main cluster (lineage I): one more highly clustered (cluster A), and another with fewer, more-distant nodes (cluster B). Cluster A and B in lineage I are separated by ≥42 mutations. The size of each node indicates the number of samples sharing a specific haplotype.

Within the two main lineages, we found several well-supported clades (Figure 1B). In lineage I, there appeared to be two well-defined clades (clusters A and B in Figures 1B and 2), which diverged approximately 158,807 years ago (95% HPD: 128,864–192,908 ya). Cluster B is only found in our Zambia collections, while members of cluster A were found in all locations. In lineage II, there were also two well-defined smaller clades with an estimated divergence time of 276,074 years ago (95% HPD: 236,527–322,922 ya; Figure 1B). The smaller clade in lineage II, containing four individuals, lacked a SNP used as a diagnostic for lineage II in a recently developed high-throughput TaqMan assay^85^. The single-SNP-based TaqMan assay targets a SNP at position 405 in the sequence of COI, where two states are considered: a T or a C^85^. This definition of lineages misidentifies ~30% of our lineage II samples as lineage I.

We constructed a network to assess the genealogical relationships between the haplotypes and to gain insight into the population level phenomena that might have contributed to the maintenance of two mitochondrial lineages in *An*. *funestus* (Figure 2). Lineage I and II are very distinct, separated by ≥137 mutational steps. On average, the lineages differ by ~160 nucleotides. Lineage II samples are separated by 77 nucleotide differences from each other on average, compared to 36 within lineage I (Table 2). Clusters A and B within lineage I are separated by ≥ 42 mutational steps. They correspond to well-supported inner clades (also clusters A and B) within lineage I in our phylogenetic analysis (Figure 1B).

### Population demography and structure

We used several population genetic statistics to test for selection or historic changes in *An*. *funestus* population size (Table 2). For the full dataset (N = 43), Tajima’s D and Fu’s Fs were not significant, and Fu and Li’s D was negative, but only moderately significant (0.10 > p > 0.05), suggesting population expansion. Neutrality analyses were also conducted for lineage I and lineage II samples separately. While lineage II did not produce significant results for any neutrality tests, lineage I was either moderately or highly significant for several statistics, again suggestive of population expansion (Table 2). Mismatch analysis (Table 2) was indicative of demographic expansion for the total sampled population, but not for lineage I or lineage II separately. The Bayesian model selection suggested a complex demographic history, and when we analyzed the data under the Bayesian Skyline model we found a signature for population expansion in the total sampled population (as well as lineage I alone) occurring approximately 80,000 years ago (Figure S4). Based on mismatch analysis, both lineage I and lineage II are consistent with models of spatial expansion.

A previous study based on partial mitochondrial gene sequences found no population structure within *An*. *funestus*^17^. Similarly, our analysis identified no clear and readily apparent geographic structure in the phylogeny of the *An*. *funestus* mitogenomes (Figure 1B). To more rigorously examine the strength of association between phylogenetic relationships and sampling locations, we used several statistical tests implemented in the BaTS package. This analysis revealed evidence for phylogenetic clustering (by country) using both the association index (p = 0.02) and the parsimony score (p = 0.01) (Table 3). The maximum clade size (p = 0.02) was significant for Zambian sequences. This suggests that the samples from Zambia were not as interspersed with samples from Tanzania or the DRC as one would expect if geography and phylogeny were randomly associated.

**Table 3:** BaTS (Bayesian Tip-association Significance testing).

Statistic	p-value
Association Index	0.02**
Parsimony Score	0.01**
MC (Zambia)	0.02**
MC (Tanzania)	0.08
MC (DRC)	1

MC = maximum clade size statistic; measures how closely particular sites are associated with monophyletic clade structure. Strength of p-value is indicated by *0.10 > p > 0.05 or **p < 0.05.

Plots of geographic distance relative to nucleotide identity are shown in Figure S5 for the total sampled population and for each lineage. Mantel tests with 1000 permutations were conducted to determine whether there was a significant relationship between genetic and geographic distance in these groups^78,86^. Both lineage I (p = 0.029*) and lineage II (p = 0.001**) had significantly related pairwise nucleotide identity and geographic distance matrices.

### Phylogenetic analysis of partial mitochondrial genes

To examine how the potential ancient population structure identified from our samples relates to the larger context of known *An*. *funestus* diversity, we constructed a maximum likelihood tree and haplotype network from a large data set including published partial mitochondrial ND5 gene sequences available from GenBank^17^ and the derived corresponding partial gene sequences from our 43 mitogenomes. The topology of the ML tree (Figure S6) as well as the haplotype network (Figure S7) again revealed a clear split between lineage I and lineage II samples. The haplotype network revealed a single, primary haplotype in lineage I containing a large number of samples from across Africa. A number of haplotypes were shared between Nigeria, Mali, and Kenya, which was reflected in the maximum likelihood tree (Figure S6). There was a large clade basal to the remainder of lineage I composed of mosquitoes from Kenya, Malawi, and Nigeria. None of our samples fell within this basal clade. Within lineage I as a whole, there was no obvious correlation of our samples with those from any other region in Africa, (Figure 1B). Samples from Mozambique fell basal to the rest of lineage II samples and tended to group apart from samples from Madagascar. Our lineage II samples fell into both groups, though samples from Madagascar appear to be more isolated within lineage II.

## Discussion

This is the first study to report the complete mitochondrial genome of *An*. *funestus* and to use complete mitogenomes to assess genetic diversity in southern and central Africa. Our data revealed higher levels of genetic diversity than previously reported using single locus markers alone. Both the Bayesian and ML trees (Figure 1B, S2) supported the co-existence of two previously-described clades, herein defined as lineage I and lineage II, in Nchelenge District, northern Zambia^23^, as well as in southern Tanzania, indicating that these lineages are more widely distributed than previously appreciated^17,23,27^. This also represents the first study to examine the distribution of lineages in southeastern DRC and extends the known distribution of lineage II in Tanzania. We have described well-supported sub-structuring within the two lineages, which may reflect much higher diversity within *An*. *funestus* than has been reported to date. Notably, our data have been shown in the context of greater African diversity, using partial ND5 sequences in a haplotype network (Figure S7). This network showed that much of *Anopheles funestus* ND5 diversity was shared across distant sites, with limited clustering by region. However, our phylogenetic clustering analysis of full mitogenomes supported the inference of geographic structure in our sample. The differing conclusions from the two datasets may be the product of either decreased homoplasy and increased phylogenetic signal of full mitogenome data, or it may be the product of a small sample of mitogenomes.

The Bayesian coalescent analyses of the complete *An*. *funestus* mitogenomes provided an estimate of the divergence times for the two mitochondrial lineages and of the clusters within lineages. Our findings were consistent with these lineages having common ancestry dating back 500,000 years, which suggests that they have evolved independently since the Pleistocene (which extended from approximately 2.58 million to 12 thousand years ago). Our divergence estimates fell on the low end of estimates from previous studies^87–91^, and specifically, our estimate of divergence between the two lineages is younger than that reported by Michel and colleagues, who used the same mutation rate (1.1–1.2% per million years) to generate an estimate of ∼850,000 years^17^.

Our *An*. *funestus* samples harbored a genomic signature of historic population expansion for the total population as well as for lineage I, though not for lineage II (Table 2). A Bayesian Skyline reconstruction (Figure S4) indicated an expansion event in the total ancestral population (3.8 to 36 million in effective population size, N_e,_) began approximately 80,000 years ago (Figure S4). Although the overall population did not reveal a signature of sudden spatial expansion (Table 2, Figure S5), mismatch analysis (Table 2) was consistent with spatial expansion for each lineage independently. Additionally, there was a significant relationship between genetic and geographic distance for both lineages independently. However, this relationship became insignificant when Tanzanian samples were removed from the analysis. Thus, these data suggested that a genetic barrier exists between our *An*. *funestus* samples, perhaps due to either the large physical distances between sampling sites or due to the Great Rift Valley, which separates our samples from Tanzania and Zambia/DRC. This latter possibility would be consistent with data from *An*. *funestus* and other related taxa across their range in sub-Saharan Africa, though our small sample size precludes eliminating the influence of extreme sampling distances. Importantly, both our identity-by-distance and Bayesian analysis of phylogenetic clustering by geography were indicative of statistical support for population structure. However, it was unclear if the weak population structure identified here is associated with the maintenance of two divergent mitogenome lineages in structured *An*. *funestus* populations, or whether historical population sizes were sufficiently large for a panmictic *An*. *funestus* population to maintain two maternal lineages.

A TaqMan assay based on COI and developed for differentiation of lineage I from lineage II^85^ based on a single SNP, failed to discriminate these lineages amongst our 43 samples. Four individuals that phylogenetically belong to lineage II (N = 14) share a nucleotide polymorphism (a ‘T’) with lineage I instead of the diagnostic SNP used in the TaqMan assay to define lineage II (a ‘C’). This finding reinforced the importance of complete mitochondrial sequences for accurate characterization of *An*. *funestus* diversity and/or revision of the assay to accurately reflect the new mitogenome data and diversity within *An*. *funestus*. We found 47 mitogenome-wide fixed SNP differences between the two lineages that may more accurately discriminate between lineages and would benefit future studies that aim to describe *An*. *funestus* lineage composition, distribution and biology in sub-Saharan Africa. A maximum likelihood tree using the partial ND5 gene of our samples along with those from Michel *et al*.^17^ (Figure S6) revealed a highly diverse sequence landscape for *An*. *funestus*, with no clear geographic clustering of our samples within the larger pan-African dataset. Taken as a whole, our data indicate that caution must be taken when using single mitochondrial genes for intra-species and population studies, due to the highly variant mitogenome of *An*. *funestus*.

## Conclusions

We have illustrated that *An*. *funestus* has a complex evolutionary history with previously undocumented levels of diversity in southern and central Africa. The diversity is ancient and geographically occurs throughout the region. We speculated that the two lineages split due to habitat partitioning in a changing African landscape during the Pleistocene, and then lineages underwent spatial expansion with consequent independent diversification. More recently the *An*. *funestus* population as a whole (predominately composed of lineage I) experienced a demographic expansion. At this time, data suggest that the lineages are at least partially sympatric. Preliminary analyses have indicated that lineages I and II may differ in habitat and/or foraging preferences (unpublished observations); ongoing work is exploring these trends. Such a difference in behavior may have important implications for vector control. Though it is tempting to theorize that lineage I and II may represent reproductively isolated populations because of the strong separation of clades in mitogenome comparisons, these results will have to be interpreted within the context of future nuclear genomics and hybridization experiments between the two lineages. Given our findings, further investigations on whether the *An*. *funestus* mitochondrial lineages represent biologically meaningful populations are warranted.

## Electronic supplementary material

Supplementary Information
