# Supplementary material for: Complete Anopheles funestus mitogenomes reveal an ancient history of mitochondrial lineages and their distribution in southern and central Africa
Source: Sci Rep. 2018 Jun 13;8:9054. doi: 10.1038/s41598-018-27092-y (PMC5997999; doi:10.1038/s41598-018-27092-y)
Supplement: Supplementary file 1 — Supplementary Information [file 41598_2018_27092_MOESM1_ESM.docx]

**Supplementary Information for**

**Complete *Anopheles funestus* mitogenomes reveal an ancient history of mitochondrial lineages and their distribution in southern and central Africa**

Christine M Jones^1^, Yoosook Lee^2^, Andrew Kitchen^3^, Travis Collier^4^, Julia C Pringle^1^, Mbanga Muleba^5^, Seth Irish^6^, Jennifer C Stevenson^1,7^, Maureen Coetzee^8,9^, Anthony J Cornel^2^, Douglas E Norris^1^, and Giovanna Carpi^1^*

^1^ Department of Molecular Microbiology and Immunology, Johns Hopkins Malaria Research Institute, Johns Hopkins Bloomberg School of Public Health, Baltimore, MD, USA

^2^ University of California at Davis, Davis, CA, USA

^3^ Department of Anthropology, University of Iowa, Iowa City, IA, USA

^4^ Daniel K. Inouye US Pacific Basin Agricultural Research Center (PBARC), Department of Agriculture, Agricultural Research Service, Hilo, Hawaii, USA,

^5^ Tropical Diseases Research Centre, Ndola, Zambia

^6^ U.S. President’s Malaria Initiative and Centers for Disease Control and Prevention, Atlanta, Georgia

^7^ Macha Research Trust, Choma, Zambia

^8^  Wits Research Institute for Malaria and Wits/MRC Collaborating Centre for Multidisciplinary Research on Malaria, School of Pathology, University of the Witwatersrand, Johannesburg, South Africa.

^9^ Centre for Emerging Zoonotic and Parasitic Diseases, National Institute for Communicable Diseases, Johannesburg, South Africa.

* Corresponding author, email gcarpi1@jhu.edu

**SI Appendix includes:**

• Figures S1 to S7

**Supplementary Figures**


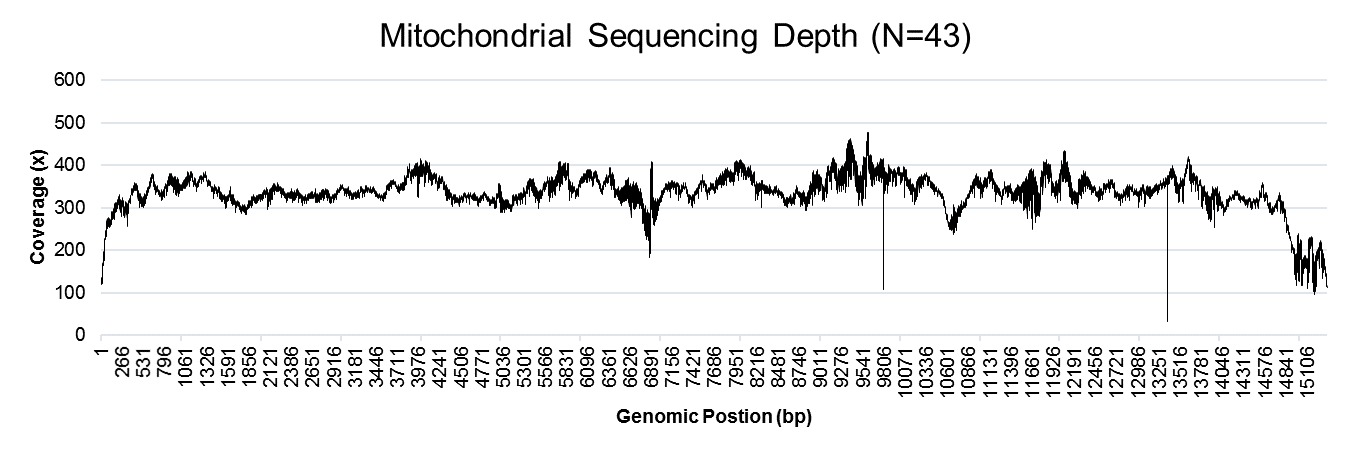


**Figure S1: Average coverage along the mitochondrial genome for 43 *An. funestus* samples.** The coverage depth is defined as the total number of sequenced bases which map to each nucleotide in the mitochondrial reference genome (AF13ICNC14-106) after removal of potential PCR duplicates and aligned reads with mapping quality below 20.


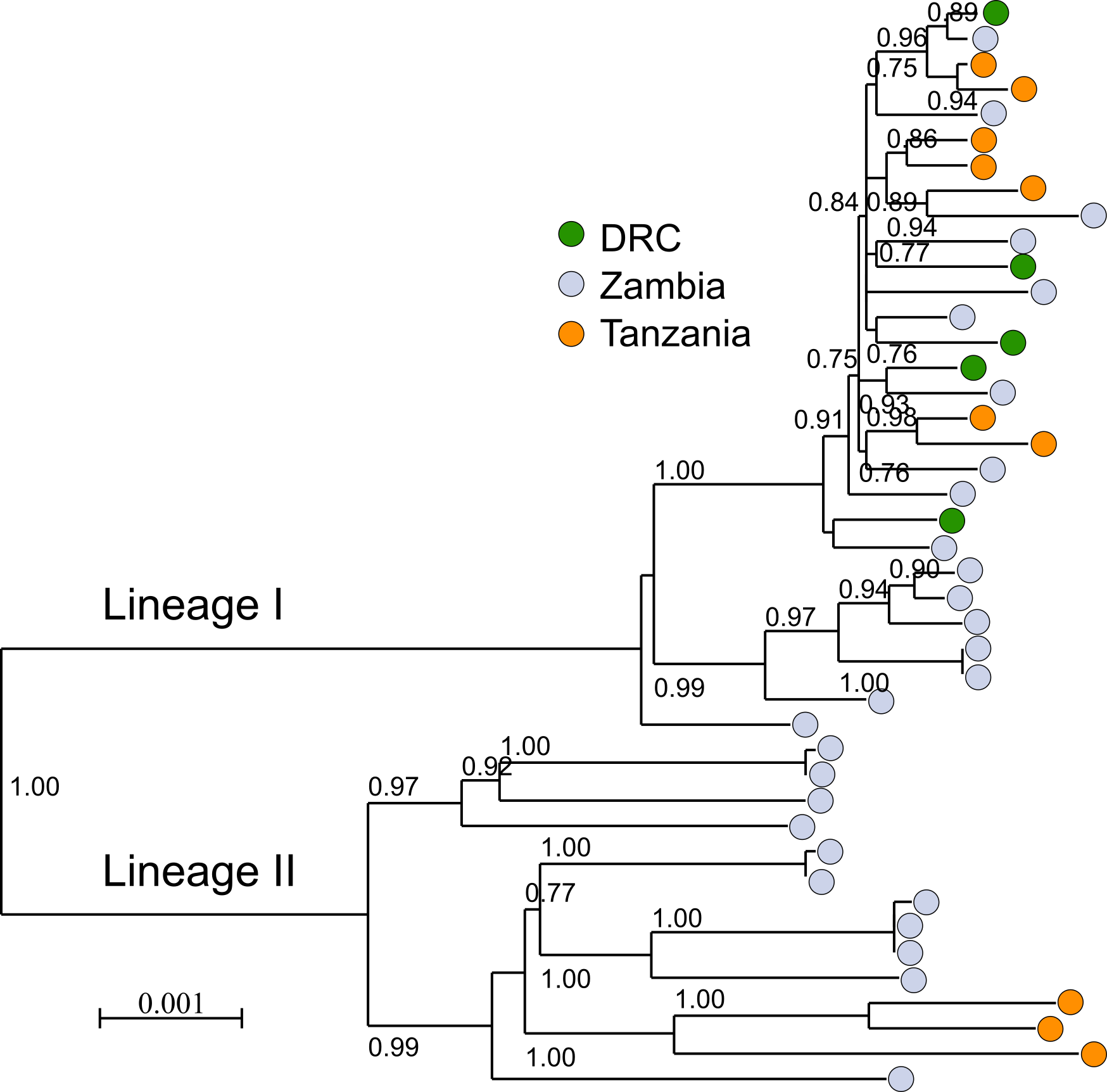


**Figure S2: Maximum Likelihood tree of 43 *An. funestus* whole mitochondrial genomes.** Maximum Likelihood tree of 43 newly sequenced *An. funestus* mitochondrial genomes using PhyML in SeaView v4 with GTR model and 1000 bootstrap replicates. Bootstrap support >0.5 shown next to appropriate nodes. Samples are colored by geographic origin, according to legend in the figure. Lineages are indicated.


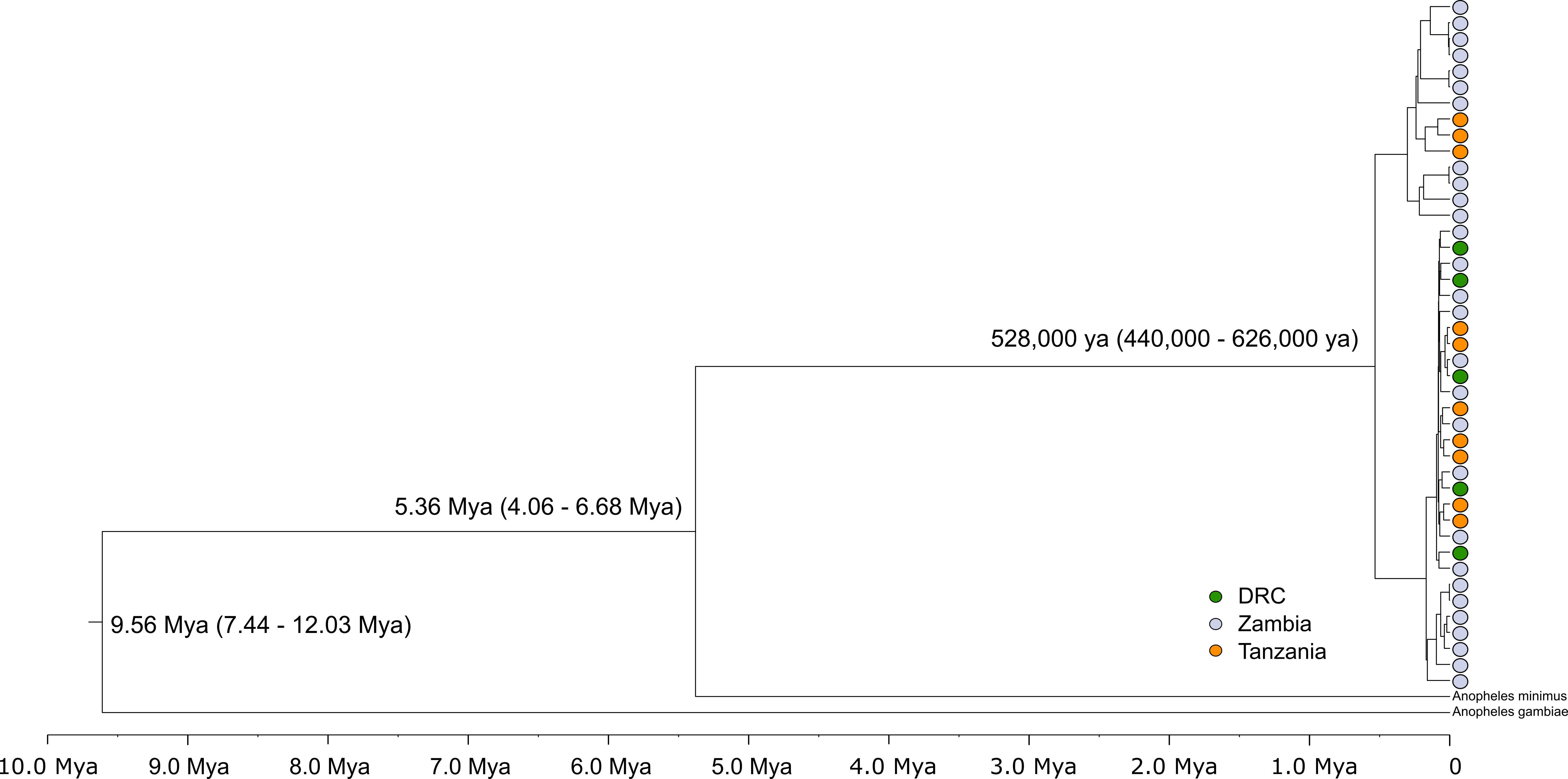


**Figure S3: Bayesian tree with *An. funestus* samples and outgroups.** Bayesian maximum clade credibility phylogeny from BEAST2 of complete mitochondrial genomes from the 43 *An. funestus* samples as well as 2 outgroups: *An. gambiae* and *An. minimus*. The model used GTR +G +I, constant population size, and a relaxed molecular clock. Samples are color-coded by geographic origin, according to the legend in the figure. Divergence dates (median estimates and 95% HPD) are given in parenthesis for major nodes. The timescale is indicated below the tree and is in year before present.


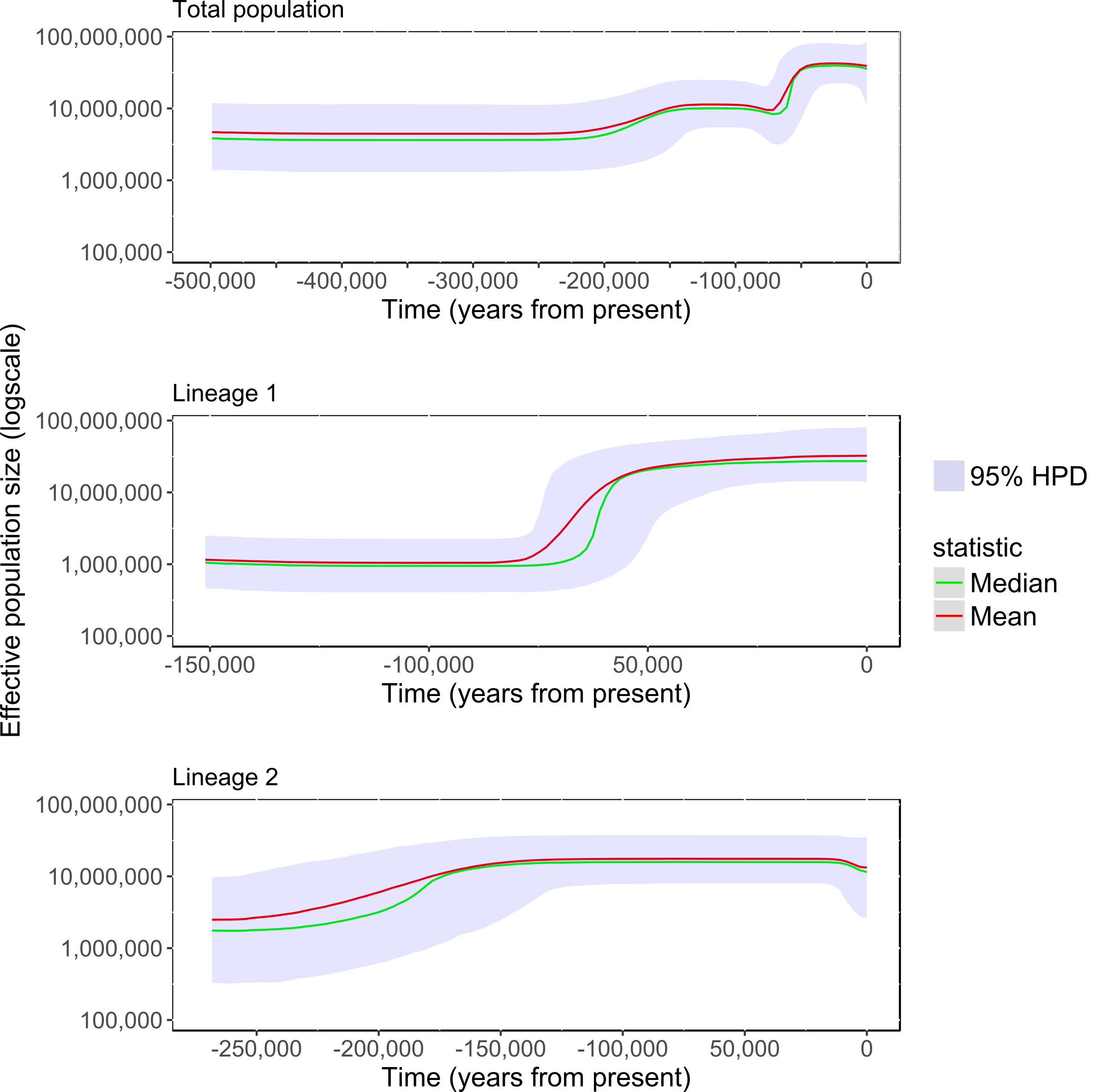


**Figure S4: Bayesian skyline plots for the total population and lineages I and II.** Bayesian skyline plot showing mean (red) and median (green) values of effective population size (95% HPD in blue). On the X axis is time going backward in years. Y axis is effective population size (log scale). Model used: GTR+G +I, relaxed clock. Top: Total population; Middle: Lineage I; Bottom: Lineage II.


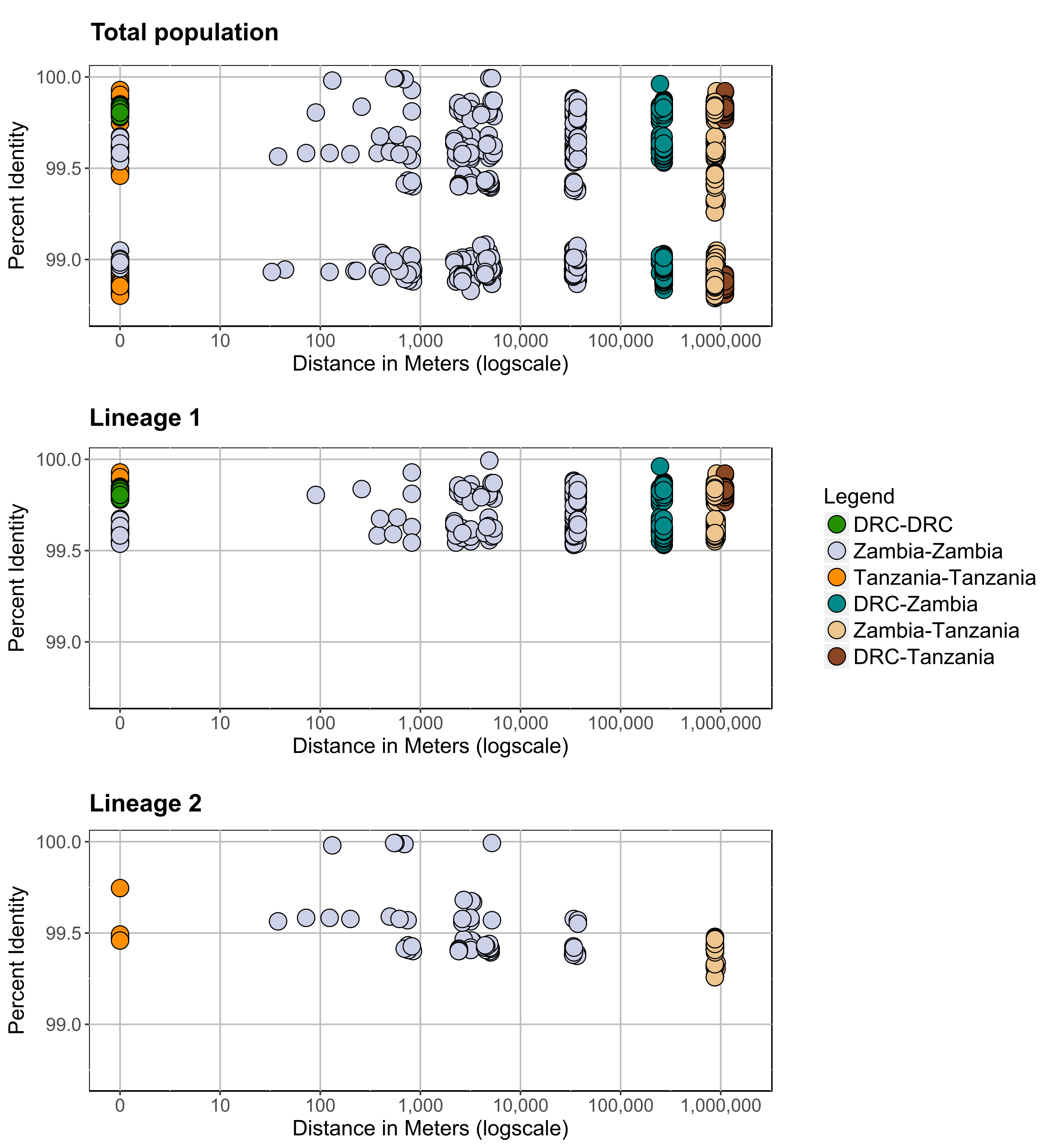


**Figure S5: Correlation between genetic and physical distance.** Pairwise genetic distance measured by nucleotide percentage identity was plotted against pairwise Euclidean distance between samples. The plots above show the relationship between the genetic and physical distance for the total population and separately for each lineage. Mantel tests were conducted on each group. There was a significant relationship between nucleotide identity and distance for lineage 1 (p = 0.029) and for lineage 2 (p = 0.001), though not for the total population. Not shown: when Tanzania samples (driving ~1 million meter comparisons) were removed from pairwise analyses, no significant correlation was seen for either lineage I (p = 0.513) or lineage II (p = 0.549).


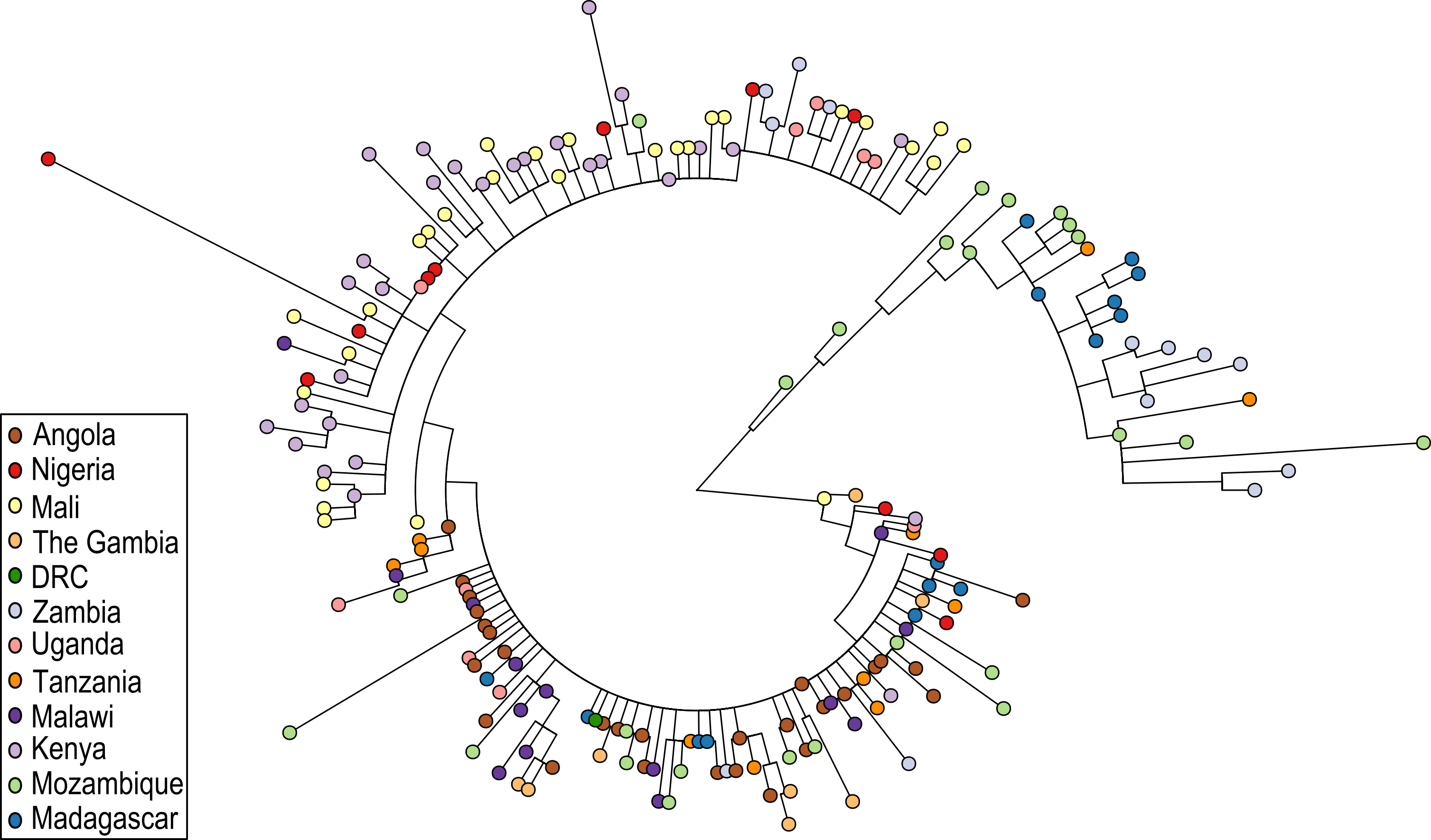


**Figure S6: ML tree of partial ND5 sequences across Africa.** Maximum Likelihood tree of the 43 *An. funestus* samples from the study in addition to 400 ND5 sequences from Michel et al. (2005) [see GenBank DQ102854–DQ103253) generated using PhyML. GTR model with 1000 bootstrap replicates. Samples are color-coded by geographic origin, as indicated in the legend on the bottom left.


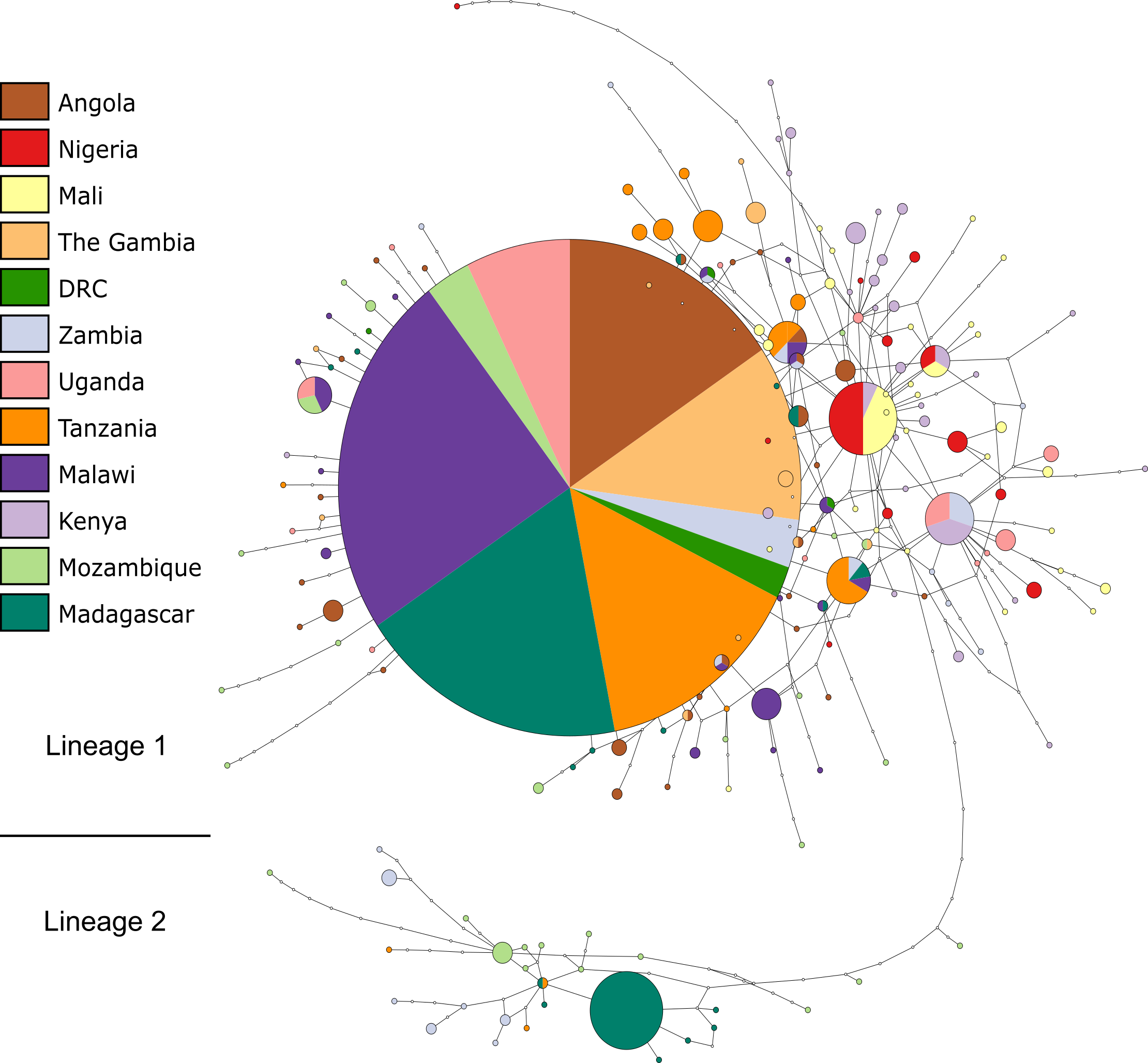


**Figure S7: Haplotype network of N=443 partial ND5 sequences.** TCS network of *An. funestus* partial ND5 sequences, including N = 43 from this study as well as N = 400 from Michel et al. (2005) [see GenBank DQ102854–DQ103253). Each circle or node indicates a haplotype, with nodes segmented and colored proportionally to the number of sequences coming from 12 African countries (see legend on left). The size of each node indicates the total number of sequences sharing that haplotype. Haplotypes are separated by small uncolored circles, each of which indicates a single mutational step. The groups representing lineage I and II samples are indicated by a dividing line on the bottom left of the figure.
